# Non–Coding RNA Performs Its Biological Function by Interacting with Macromolecules

**DOI:** 10.3390/ijms242216246

**Published:** 2023-11-13

**Authors:** Yao Li

**Affiliations:** State Key Laboratory of Genetic Engineering, School of Life Science, Fudan University, Shanghai 200433, China; yaoli@fudan.edu.cn

One of the most significant discoveries resulting from the sequencing of the human genome is the realization that a large portion (over 85%) of the genome is transcribed into RNA, yet less than 2% of it encodes protein-coding genes. This non-coding RNA (ncRNA) constitutes several groups, including well-known examples like tRNA and rRNA, as well as small RNA, long non-coding RNAs (lncRNAs), and circular RNA (circRNA). Among these, lncRNAs are the most abundant group of non-coding RNAs produced by the genome. While many newly discovered ncRNAs are yet to be fully characterized and validated, it is believed that a significant proportion of them serve important functions—yet many of these remain unclear.

The functions of ncRNAs are often influenced by factors such as their localization, sequence, and secondary structure. They can regulate protein expression and activity at various levels, leading to a diverse array of biological effects. Regardless of the specific type of ncRNA, their mechanism of action primarily involves interactions with macromolecules, including DNA, RNA, and proteins.

## 1. RNA–DNA Interaction

There is increasing evidence supporting the interaction of long non-coding RNAs (lncRNAs) with DNA, as well as their role as transcriptional activators or repressors in gene regulation. LncRNAs have the ability to target specific regions of the genome for epigenetic modifications. They can directly bind to purine-rich double-stranded DNA sequences through Hoogsteen base pairing, forming RNA–DNA triplex structures [1]. These classical triplex structures are categorized into pyrimidine motifs, purine motifs, and mixed motifs, depending upon the base composition of the RNA strand [2]. However, it is important to note that the formation of triplexes between pyrimidine-rich RNA strands and DNA is hindered in biological systems due to the requirement of an acidic pH for the protonation of cytosine. Therefore, under normal biological conditions, triplexes are more likely to form between purine-rich RNA strands and DNA double strands. 

Bioinformatics analysis has been utilized to predict the existence of numerous triplex-forming motifs in the human genome. Interestingly, these motifs tend to accumulate in gene regulatory regions, particularly in the promoter region [3]. This suggests that lncRNAs can form triplex structures that have the potential to upregulate or downregulate gene expression, either in a cis-acting manner (affecting neighboring genes) or in a trans-acting manner (affecting distant genes). However, it is worth noting that the direct binding of lncRNAs with DNA in experimental settings is rarely demonstrated [4]. 

The RNA–DNA triplex structure can regulate gene transcription in two main ways. Firstly, it can increase chromatin accessibility and facilitate transcription by altering chromatin conformation. Although this mode of regulation has been suggested based on bioinformatics analysis and preliminary experimentation, more in-depth experimental evidence is needed. Secondly, the RNA–DNA triplex structure can recruit transcription factors or epigenetic modulators, thereby influencing gene transcription in both directions. Two independent studies have shown that the vast majority of lncRNAs positively regulate gene expression through RNA–DNA triplexes, suggesting that the first mode of regulation is more predominant [3,5].

In our previous study, we proposed that the stable triplex structure formed by AP006284.1 lncRNA could alter chromatin conformation and promote the transcription of target genes. We hypothesized that this stable triplex structure weakens the interaction between DNA and histones, increasing the accessibility of chromatin and enhancing transcription [6]. It is worth noting that enhancer RNAs (eRNAs) play a crucial role in regulating chromatin conformation and transcription activation [7], and it is possible that RNA–DNA triplexes contribute to their enhancer function.

Studies have also shown that RNA–DNA triplex structures can recruit chromatin-modifying enzymes or affect chromatin remodeling. For example, the lncRNA PARTICLE forms a triplex structure with the promoter region of MAT2A, recruiting methyltransferases and polycomb repressor complexes to inhibit MAT2A transcription [8]. Another example is lncRNA-Khps1, which forms triplexes with the promoter region of SPHK1 and recruits effector proteins to activate SPHK1 transcription through local changes in the chromatin structure [9]. 

Overall, the RNA–DNA triplex structure represents a fascinating mechanism by which lncRNAs can regulate gene expression. Further experimental studies are necessary in order to fully elucidate the functional significance and mechanisms of RNA–DNA triplexes in gene regulation.

## 2. RNA–RNA Interaction 

ncRNAs are also involved in post-transcriptional regulation through RNA–RNA interactions, where they interact based on the principle of Watson–Crick base pairing. ncRNAs regulate mRNA expression through various mechanisms, including affecting mRNA stability, splicing activity, modifications, capping, and translation efficiency. For example, lncRNAs can act as miRNA sponges, indirectly derepressing the expression of mRNAs targeted by the miRNAs. Additionally, ncRNAs can modulate translation by binding to ribosomes or mRNA transcripts during translation.

One of the most well-known RNA–RNA interactions is the interaction between small RNAs, such as siRNAs and miRNAs, and the RNA-mediated silencing pathways. These interactions have shed light on eukaryotic gene regulation and revealed novel host defenses against viruses and transposons. MicroRNAs (miRNAs) are small, non-coding RNAs containing about 23 non-coding nucleotides that downregulate protein-coding transcripts by base pairing with their target mRNAs. They play crucial roles in developmental and pathological processes in both animals and plants [10]. miRNAs are involved in various physiological processes, including development, cell death, and cell signaling. They also contribute to the pathogenesis of different diseases. 

miRNAs are typically incorporated into the RNA-induced silencing complex (RISC), where they guide the complex to target mRNAs through base pairing. AGO proteins in the RISC complex recruit factors that induce translational repression, mRNA deadenylation, and mRNA decay [11]. miRNA-binding sites are often found in the 3′ untranslated region (UTR) of mRNAs, and it is estimated that more than 60% of human protein-coding genes contain at least one conserved miRNA-binding site. siRNAs can also form RISC complexes that bind to any part of the mRNA and degrade it in an exact pairwise manner.

Another important RNA–RNA interaction is competitive endogenous RNA (ceRNA), which was first proposed by Salmena and colleagues [12]. ceRNAs refer to all types of transcripts, including mRNA, tRNA, rRNA, lncRNA, pseudogene RNA, and circular RNA, as they can all be targeted by miRNAs depending on the spatiotemporal context. Most ceRNAs contain potential microRNA response elements (MREs), share common miRNAs, and compete for binding with miRNAs [12]. Pseudogene RNAs can act as sponges by competitively binding common miRNAs, thereby releasing or attenuating miRNA-mediated repression by sequestering miRNAs away from their parental mRNAs. LncRNAs, as a major type of ncRNAs, can act as molecular sponges by competitively targeting miRNAs, thereby attenuating the miRNA-mediated degradation or inhibition of their own downstream protein-encoding target genes. This regulation mechanism allows lncRNAs to participate in a variety of physiological and pathological processes [13].

Overall, RNA–RNA interactions play crucial roles in post-transcriptional regulation, with ncRNAs acting as important mediators in these interactions.

## 3. RNA–Protein Interaction

RNA–protein interactions are a common phenomenon that plays a crucial role in the cellular functions of non-coding RNAs (ncRNAs). Understanding the molecular mechanisms underlying the assembly and regulation of ncRNA–protein complexes is essential for comprehending their cellular functions. Compared to proteins, lncRNAs are generally larger, more complex, and prone to intermolecular interactions. Due to their length, ncRNAs can engage in both short- and long-range interactions, resulting in complex folded structures and the recruitment of various proteins.

On the one hand, proteins play crucial roles in regulating RNA transcription, processing, and stability in order to ensure proper gene expression. They are involved in multiple steps of RNA metabolism, contributing to the intricate network of gene regulation. On the other hand, it is important to note that ncRNAs also have an impact on protein function. They interact with proteins through their complex tertiary structures, acting as scaffolds or molecular chaperones. These interactions play a pivotal role in the recruitment and assembly of protein complexes, as well as the regulation of their activities. As a result, ncRNAs are able to perform a wide range of biological functions and contribute to various cellular processes [14]. 

The proteins that ncRNAs regulate are distributed throughout the nucleus and cytoplasm. In the nucleus, ncRNAs can act as scaffolds for transcription factors or chromatin remodeler proteins, regulating transcription or chromatin accessibility, respectively (*trans*–regulation). In the cytoplasm, they can affect protein assembly and activity by forming complexes with proteins, participating in various biological processes such as signal transduction and enzyme activity. For example, Huarte’s research revealed that LincRNA-p21 can repress transcription by physically associating with hnRNP-K, which is required for the proper genomic localization of hnRNP-K at repressed genes and regulates p53-mediated apoptosis [15]. Jiang’s study reported that DEANR1 facilitates FOXA2 activation by facilitating SMAD2/3 recruitment to the FOXA2 promoter [16]. 

Additionally, ncRNAs have been found to act as sponges for RNA-binding proteins (RBPs), recruiting and sequestering RBPs, inhibiting their activity, or altering their localization in the cytoplasm and nucleus [17]. The association of ncRNAs with RBPs forms lncRNA–protein complexes that play roles in a wide range of biological processes.

In recent years, there has been a growing recognition of the role of proteins in undergoing phase separation and phase transition, which are involved in important physiological and pathological processes. RNA, although traditionally considered a “supporting player,” has the ability to influence the phase transition of proteins through its interactions with them. While RNA has mostly been studied in its supportive role in phase separation and phase transition, it has the potential to actively drive these processes. A recent study by Lu and colleagues has shed light on the ability of RNA molecules with expanded CAG repeats (eCAGr) to undergo sol–gel phase transitions. This cytoplasmic RNA gelation process sequesters eEF2, leading to disruptions in global protein synthesis. This finding highlights the potential of RNA to actively participate in the phase separation and phase transition processes, exerting a significant impact on cellular activities [18].

These insights demonstrate that RNA’s involvement in phase separation and phase transition is not limited to a mere supporting role. It possesses the potential to drive these processes and play a more significant role than has been previously recognized. Further research in this area will help us to better understand the intricate interplay between RNA, proteins, and phase separation, contributing to our knowledge of cellular function and dysfunction.

In summary, ncRNAs regulate diverse biological processes and play regulatory roles in various biological processes and diseases. They achieve their functions through interactions with DNA, RNA, and proteins. LncRNAs can regulate gene expression at both the transcriptional and post-transcriptional levels, and participate in the formation and activity regulation of protein complexes. The interplay between ncRNAs and macroproteins is vital for organism development and the coordination of biological processes, including metabolism, the maintenance of genome integrity, immune responses, and disease and stress responses.
